# Mediation role of cardiorespiratory fitness on association of physical activity and physical literacy among 8–12 years old children: the PAK-IPPL cross-sectional study

**DOI:** 10.3389/fped.2024.1383670

**Published:** 2024-09-13

**Authors:** Syed Ghufran Hadier, Liu Yinghai, Liu Long, Syed Danish Hamdani, Syed Muhammad Zeeshan Haider Hamdani

**Affiliations:** ^1^ School of Physical Education, Shanxi University, Taiyuan, Shanxi, China; ^2^ Department of Sports Sciences, Bahauddin Zakariya University, Multan, Pakistan; ^3^ Department of Physical Education, Suzhou University, Suzhou, Anhui, China; ^4^ Division of Olympic Sports, China Swimming College, Beijing Sport University, Beijing, China; ^5^ School Education Department, Government of Punjab, Multan, Pakistan; ^6^ Department of Sports Sciences, Faculty of Social Science, Bahauddin Zakariya University, Multan, Punjab, Pakistan

**Keywords:** public health, cardiorespiratory fitness, childhood adiposity, physical activity, physical literacy, mediation analysis, South Punjab Pakistan

## Abstract

**Background:**

The effect of cardiorespiratory fitness (CRF) on the relationship of physical activity (PA), and physical literacy (PL) in 8–12 Pakistani children are largely unknown. Therefore, this study aims to examine the mediating role of CRF in the relationship between PA and PL in this demographic.

**Methods:**

This cross-sectional study involved 1,360 children aged 8–12 (mean age = 10.00, SD = 1.41 years) from 85 higher secondary schools in South Punjab, Pakistan. Data were collected during the 2020–2021 academic year using the Canadian Assessment of Physical Literacy-2 (CAPL-2) protocol to assess physical activity (PA), Body Mass Index (BMI), and PL levels. CRF was measured using the PACER (Progressive Aerobic Cardiovascular Endurance Run) test. Descriptive statistics, independent samples *t*-tests, Chi-squared tests, and Generalized Additive Models for Location, Scale, and Shape (GAMLSS) were used for age and sex-specific CAPL-2 scoring. Associations among components were evaluated through Pearson's correlation, multivariate logistic regression, and mediation analyses.

**Results:**

The study revealed that boys had significantly higher scores in CRF, PA, and PL across all age groups compared to girls (*p* < 0.001), with boys' scores being 20%, 10%, and 14% higher, respectively, than those of girls. Conversely, overweight children showed significantly reduced PA and PL levels (*p* < 0.001). An inverse correlation was found between BMI and CRF (*r*^2^ linear = 0.022; quadratic = 0.028). CRF scores had significant negative correlations BMI (*r* = −0.143) and positive associations with MVPA, PA, DB, and PL (*r* ranging from 0.241 to 0.624). CRF was observed to partially mediate the association between MVPA and PL. The direct impact of PA on PL was significant and meaningful (*β* = 0.002, *p* < .001). Additionally, the indirect effect of PA on PL through CRF was also significant (*β* = 0.001, *p* < .001), indicating that CRF serves as an important mediator in this relationship. The combined total effect of PA on PL, which includes both direct and mediated pathways, was robust and highly significant (*β* = 0.003, *p* < .001).

**Conclusion:**

The study revealed a strong positive correlation between CRF, PA, and PL, but a negative one with BMI in South Punjab children aged 8–12. Notably, CRF and PA emerged as significant predictors of PL levels in this population. Consequently, interventions that are both systematic and targeted towards improving these factors should be implemented as strategies to enhance children's PL levels and promote physically active behaviors.

## 1 Introduction

The escalating prevalence of childhood obesity and physical inactivity constitutes a significant public health concern, both globally and in Pakistan, which ranks tenth worldwide in obesity rates (1). Alarmingly, projections indicate that by 2030, obesity may affect 5.4 million school-aged children in Pakistan (2), highlighting the challenges of obesity and sedentary lifestyle. This alarming trend emphasizes the urgent need to explore effective strategies to combat issues such as childhood obesity, physical inactivity, and their associated risks, including an increased prevalence of chronic diseases.

To address these correlated public health challenges, it is essential to understand the role of Cardiorespiratory Fitness (CRF), recognized as a strong indicator of general health and well-being in both children and adults (3). Research indicates that higher CRF levels, associated with lower body mass index (BMI) in children, correlate with a reduced propensity towards metabolic syndrome compared to their inactive and obese peers (4). This finding emphasizes the importance of fostering fitness to mitigate obesity and its long-term health implications from childhood onwards.

CRF emerges as a critical factor, with evidence indicating the correlation between regular physical activity (PA), improved cardiorespiratory health, and overall well-being in children (3). This relationship stresses the critical need for an assessment of children's PA patterns and fitness levels, particularly their implications for CRF, PA, and physical fitness, to combat the epidemic of obesity and inactivity (5, 6). Despite the evident challenges in formulating effective strategies for obesity management and PA enhancement, the emerging concept of Physical Literacy (PL) gained prominence as a potential solution (7). Defined by the International Physical Literacy Association (2014) as “the motivation, confidence, physical competence, knowledge and understanding to value and take responsibility for engagement in physical activities for life” (8), PL offers a viable solution to the dual scourges of inactivity and obesity. It promises a pathway to foster lifelong PA and long-term healthy lifestyle, especially among children at heightened risk due to increased adiposity and diminished fitness levels (5, 9).

Recent studies have demonstrated a clear association between childhood obesity and impaired physical performance (10). Obese children often exhibit lower levels of physical fitness, which, in turn, affects their ability to engage in physical activities effectively (11). This impairment can lead to a vicious cycle where decreased physical activity levels further exacerbate obesity and associated health issues (10, 12). Understanding this relationship between obesity and physical performance is crucial for developing comprehensive interventions that address both aspects simultaneously.

The complex relationship between the high prevalence of obesity, and physical inactivity among children in Pakistan and by extension, globally demands of a focused understanding of poor CRF's role (13). Enhancing CRF through moderate to vigorous physical activity (MVPA) might offer an effective strategy for addressing these conditions (14). By focusing on the mediating role of CRF, this study aims to delve deeper into the relationship through which physical activity influences PL and, consequently, overall health (14). This approach could reveal critical strategies for enhancing healthy outcomes in children by demonstrating how improvements in CRF can lead to better PL, thus promoting more active and healthier lifestyles (9).

The emphasis on children aged 8–12 years is particularly judicious, given the formative nature of this developmental stage for adopting and solidifying healthful behaviors. Insights from studying this age group are critical for designing age-appropriate interventions that promote physical activity, improve CRF, and enhance PL, thereby fostering environments and programs that encourage a more active lifestyle. This study is instrumental in creating environments and programs that incentivize a more dynamic lifestyle, crucial for obviating obesity and improving general health. In the specific context of Pakistan, where cultural, social, and infrastructural factors may impact physical activity and wellbeing (15), a focus on PL through the lens of enhanced CRF offers a culturally sensitive, sustainable approach to navigating the public health challenges posed by obesity and physical inactivity.

Moreover, the correlation between obesity, poor motor coordination & performance, decreased CRF, and the escalating global trend of physical inactivity necessitates an in-depth investigation into the relationship between CRF, PA, and PL in Pakistani children (10). To date, no study has examined these correlations within this demographic, particularly focusing on the southern Punjab region of Pakistan or broadly in South Asia (9). The Pakistan Initiative to Promote Physical Literacy (PAK-IPPL) study offers a unique opportunity to analyze these associations in a large sample of Pakistani children. Utilizing the Canadian Assessment of Physical Literacy- second edition (CAPL-2), this study aims to identify associations so that domains needing improvement in PL can be identified, and targeted interventions can be developed (16). This study aimed to fill the existing research gap by exploring the mediating role of CRF in the relationship between PA and PL, potentially identifying the need for a targeted approach to improving child health outcomes and contributing to the mitigation of the obesity and physical inactivity epidemic.

Therefore, This study aims to explore the associations between physical activity and physical literacy, alongside body mass index and each PL domain among 8–12-year-old school children in south Punjab Province, Pakistan. Additionally, it aims to investigate the extent to which cardiorespiratory fitness, measured by the Progressive Aerobic Cardiovascular Endurance Run (PACER), mediates the association mediates the association between PA and PL.

We hypothesize that CRF significantly mediates the relationship between PA and PL in this age group. Specifically, higher levels of PA are expected to enhance CRF, which in turn, is anticipated to positively influence PL outcomes. The hypothesis that CRF significantly mediates the relationship between PA and PL is supported by existing research, which consistently demonstrates the mediating role of CRF in the relationship between physical activity, obesity and various health and cognitive outcomes (11, 17). By investigating these relationships, our study aims to provide comprehensive insights into how enhancing CRF can lead to better physical literacy and activity levels among school children.

**Figure 1:**
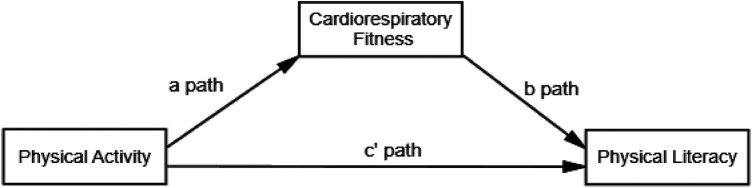
Hypothesized model for the association between the study variables. The figure illustrates the theorized model with physical literacy serving as an outcome.

**H1**: CRF mediates the relationship between PA and PL level of 8–12-year-old school children. Figure 1 illustrates the relationship between models.

This study will yield critical insights for public health initiatives, informing the development of targeted interventions and educational strategies aimed at improving health and physical education programs in schools. Moreover, the findings will serve as a valuable resource for healthcare providers, educators, and policymakers dedicated to fostering healthier lifestyles among children.

## 2 Methods

### 2.1 Study design and sampling

This cross-sectional study was conducted in South Punjab province of Pakistan, focusing on three divisions: Multan, Bahawalpur, and Dera Ghazi Khan. A stratified random sampling method was employed to ensure representativeness across both geographical and demographic dimensions within the selected province (18, 19). The sample size was calculated using Cochran's formula: $n\;=\;\frac{{\left(Z\right)}^{2}\mathrm{PQ}}{e^{2}}\;\times \;D$ (20). In this formula, Z represents the standard normal distribution, which is 1.96 at a 5% significance level. The expected proportion (P) is 0.23, while Q is 1 - P (0.766), the level of precision e^2^ is 0.002545, and D, the design effect, is set to 5. This resulted in a calculated sample size of approximately 1,360 participants.

Initially, 87 higher secondary schools were selected, with 29 schools from each division using an equal allocation method. However, two schools declined participation, resulting in a final sample of 85 schools. From each school, 16 students aged 8–12 years were randomly selected, achieving a total of 1,360 participants. However, an 11% dropout rate was observed, primarily due to participants' refusal or inability to adhere in completion of all the test included in the CAPL-2 protocol. To address this attrition and maintain the integrity of the study's sample size, additional participants were sourced from the same demographic pool. The final sample distribution included 455 participants from Multan, 455 from Bahawalpur, and 450 from Dera Ghazi Khan, ensuring a balanced representation across the selected divisions. Stratified random sampling with equal allocation ensured an equitable distribution across schools, ages, genders, and cities, as detailed in a previous study (21).

### 2.2 Ethics approval

Ethical approval for this study was obtained from the School of Physical Education, Shanxi University in 2020 (Reference No: SXULL201912), adhering to the principles of the Declaration of Helsinki. Additional approvals were secured from relevant authorities in Pakistan. Bahauddin Zakariya University, Multan, granted ethical approval (Letter No: 374/UREC/2020), and permission was obtained from the South Punjab Education Department (Letter No: 2189/GB). Participation in the study was voluntary, requiring verbal assent from all students and written consent from a parent or guardian. Permission to use the CAPL-2 questionnaire and related materials was obtained from the Healthy Active Living and Obesity (HALO) research group via email correspondence. Data collection commenced following these approvals.

### 2.3 Data collection procedures

This study was conducted during the 2020–2021 academic year, commencing on September 12, 2020, and concluding data collection on April 17, 2021. The methodology encompassed the use of valid and reliable questionnaires (22) and various assessment tools to gather information on demographic and anthropometric variables, weight status, cardiorespiratory fitness, physical activity, and Physical Literacy.

Before data collection, a proper method and schedule were established. The evaluation of physical literacy involved designated research teams for each district, coordinated by a principal investigator. Each team included a sports science faculty member from Bahauddin Zakariya University (BZU) as the lead and four final semester MSc sports sciences students from BZU serving as data collectors. Female-only teams were assigned to female schools.

All team members participated in a preparatory workshop held at BZU, where they received comprehensive training on the CAPL-2 protocol through instructional videos and manuals, both in hard and soft copy (details at https://www.activehealthykids.org/capl-2-training-materials/). The principal investigator addressed queries, provided additional guidance, and oversaw the training on test administration in the field. This preparatory phase ensured that all evaluators were well-versed in the testing procedures and timelines for data collection.

#### 2.3.1 Data collection phase

To ensure consistency and efficiency, data collection occurred consistently across all schools. A three-day standardized protocol was adopted.

**Figure 2:**
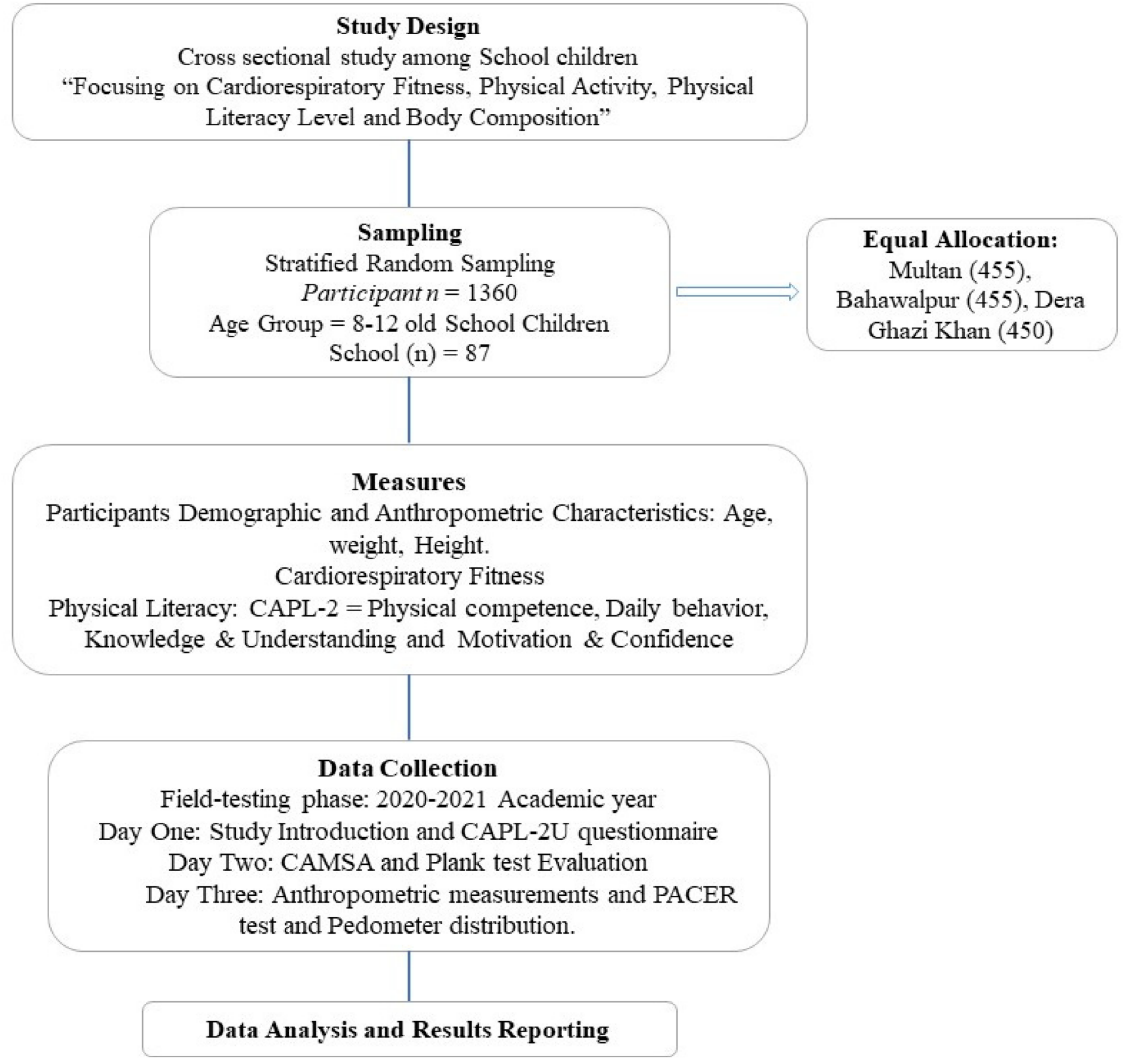
Flow chart of study procedure.

**Day 1**: Introduction and Questionnaires: Participants were briefed on the study's objectives and introduced to the concepts of Physical Literacy, Physical Activity, and Cardiorespiratory Fitness. Participants completed the CAPL-2 Urdu version of the questionnaire, which assessed the daily behavior domain (Physical Activity) along with other domains (22).
**Day 2**: Performance Assessments: A five-member assessor team evaluated the students' performance on the Canadian Agility and Movement Skill Assessment [CAMSA (23)] and the plank test (24). Each test procedure was demonstrated to the students, who were then allowed one practice attempt for the plank test and two for the CAMSA.
**Day 3**: Anthropometric Measurements and Endurance Test: On the final day, anthropometric measurements were taken, and the 20 m shuttle run test also known as the Progressive Aerobic Cardiovascular Endurance Run (PACER) test was administered to assess cardiovascular endurance (25). Subsequently, students were given pedometers, with instructions, and tracking logs to record their activity. Arrangements were made to accommodate absentees and address missing pedometer data as per the guidelines in the CAPL-2 manual. The procedural flow of the study is illustrated in Figure 2.

### 2.4 Measurers

#### 2.4.1 Demographics and anthropometrics

Student's age, sex and birth year were obtained. Height measurements were conducted using a Digital Electronic Height-Weight Measurement Scale, with students standing barefoot, maintaining a straight posture against a level surface. Weight, measured in kilograms on the same scale, required the removal of shoes, excess clothing, and accessories, ensuring accuracy to 0.1 kg.

Body Mass Index (BMI) was calculated using the CDC-endorsed standard formula $\mathrm{BMI}\;=\;\frac{W(\mathrm{kg})}{Hm^{\left(2\right)}}$ was employed in this study for BMI calculation and assessment (26, 27). For body weight classification, CDC percentile classifications were used: below the 5th percentile was categorized as underweight, between the 5th and 85th percentile as normal weight, at or above the 85th percentile as overweight, and at or above the 95th percentile as obesity (21, 26).

Waist circumference (WC) and hip circumference (HC) were measured with standard precision. For WC, a standard non-elastic flexible measuring tape was placed approximately 1 cm above the navel, ensuring measurements were accurate to one decimal place in centimeters. Participants were positioned upright, with arms extended laterally, feet together, and instructed to breathe normally without intentionally contracting abdominal muscles or holding their breath, to allow for an even weight distribution across both feet. The examiner, stood in front of the participant, accurately identified the horizontal plane of the navel to within 1 cm. The examiner needed to maintain an eye-level alignment with the scale on the lower edge of the tape to guarantee measurement accuracy. The tape was kept parallel to the ground, and measurements were recorded to the nearest 0.1 cm. HC measurements were conducted at the fullest part of the buttocks using a similar standard tape. This approach ensured the uniformity of the measurement process and the accuracy of the recorded data.

#### 2.4.2 Physical literacy

Physical literacy encompasses the skills, knowledge, and behaviors that enable individuals to confidently and effectively engage in physical activities throughout their lives. It is a holistic concept that includes cognitive, behavioral, physical, and social dimensions of physical activity and a healthy lifestyle. Promoting lifelong participation in physical activities, physical literacy is crucial for overall health and well-being.

**Figure 3:**
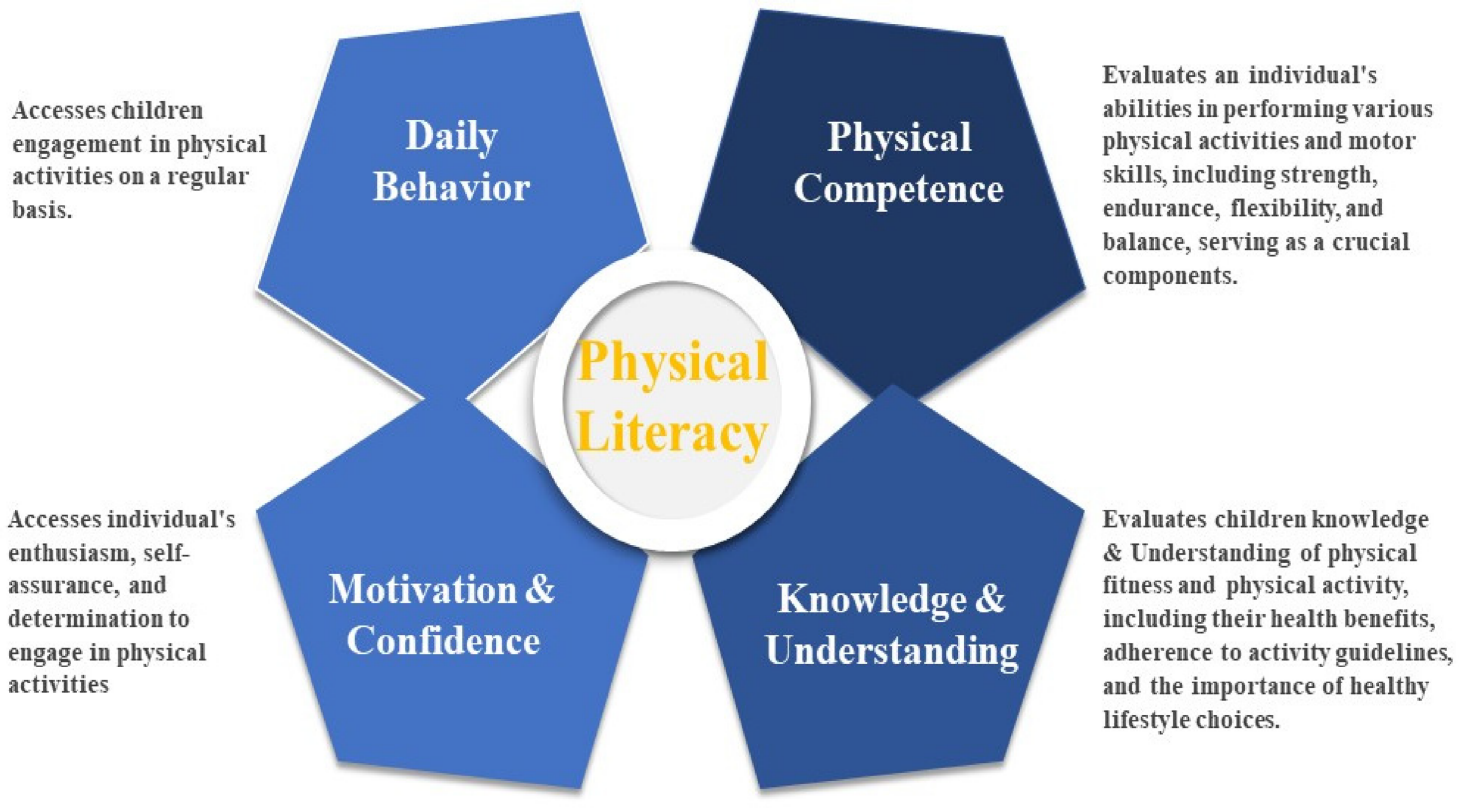
Physcial literacy and sub domains.

The Canadian Assessment of Physical Literacy-2, introduced in 2015 and validated in various international contexts, is an effective evaluative instrument designed to assess the complex dimensions of physical literacy in children (16). CAPL-2 examines physical literacy through four interconnected domains: Physical Competence (PC), Motivation and Confidence (M&C), Knowledge and Understanding (K&U), and Daily Behavior (DB) (8, 16). These domains, while distinct in focus, are interrelated and mutually reinforcing. For example, a child's understanding of the benefits of physical activity can enhance their motivation, and their daily behaviors often reflect their physical competencies (16). This study utilized the Urdu version of CAPL-2 (22). Figure 3 illustrates the domains of physical literacy.

##### 2.4.2.1 Physical competence

The PC domain was evaluated using a 30-point scoring system, assessing cardiovascular endurance (PACER 20 m Shuttle Run), muscular strength (Plank Test), and motor skills (Canadian Agility and Motor Skill Assessment-CAMSA), each contributing a maximum of 10 points. CAMSA involves the development of a wide range of fundamental movement skills and the ability to apply these skills in various contexts. It includes skills such as running, jumping, throwing, and balancing, which are foundational for more complex physical activities and sports. These assessments provide insights into a child's physical capabilities and areas where they may need improvement. The scoring was based on the performance criteria outlined in the CAPL-2 manual (6).

#### 2.4.3 Cardiorespiratory fitness

In this study, the 20-m shuttle run test (20mSRT), also known as the Progressive Aerobic Cardiovascular Endurance Run (PACER), was employed as a valuable marker of the Physical Competence domain (28). The 20mSRT is a well-validated measure of cardiorespiratory fitness among children and is favored for its ease of administration, minimal equipment requirements, and suitability for assessing large groups of children (29). Previous research has shown a positive correlation between 20mSRT performance and various aspects of physical literacy, including physical fitness, daily physical activity, cognitive ability, and psychosocial health (3, 29).

##### 2.4.3.1 Knowledge and understanding

This domain was assessed through questionnaires evaluating children's knowledge of physical activity and health concepts. The assessment covered awareness and understanding of health-related fitness, the benefits of physical activity, and how to engage in physical activities safely and effectively. It also included understanding the rules and strategies of various physical activities and sports. The K&U domain was evaluated using multiple-choice and fill-in-the-blank questions, designed to estimate children's knowledge of physical activity recommendations, fitness, and strategies for improving physical competence. This domain contributed 10 points to the overall Physical Literacy scores (16).

##### 2.4.3.2 Motivation and confidence

Motivation and confidence refer to the willingness to participate in physical activities and the confidence in one's ability to perform physical tasks. This component is crucial for sustaining engagement in physical activity, as motivated and confident individuals are more likely to be active regularly. M&C were evaluated through self-report questionnaires that gauged a child's enjoyment of physical activity, their perceived competence in performing physical tasks, and their motivation to participate in various physical activities. This assessment helped to understand the psychological and emotional factors influencing a child's physical activity behaviors. The M&C domain, with a total of 30 points, probed four sub-constructs, each contributing 7.5 points (6). These constructs encompassed intrinsic motivation, perceived competence regarding physical activity, predilection, and adequacy.

##### 2.4.3.3 Daily behavior domain

The Daily Behavior domain evaluates regular engagement in physical activities, including both structured activities such as sports and exercise, and unstructured activities like playing, walking, and active commuting. It reflects the integration of physical activity into a child's daily routine by capturing the frequency, intensity, and duration of their physical activities. The DB domain contributes 30 points to the composite Physical Literacy scores and evaluates children's physical activity levels using two methods: objective measurement through daily step counts and subjective measurement through moderate to vigorous physical activity (MVPA) (8).

Objective measurement was conducted using pedometers (DigiWalker SW-200, Yamax Corporation, Tokyo, Japan) to count daily steps. Participants were provided with pedometers to wear above their right hip bone, attaching them to their waistbands of pants for boys and shalwar for girls for seven consecutive days. The first day served as a “practice day” to familiarize them with the device. For the following six days, participants or their guardians recorded the daily step count before bedtime, noting any instances when the pedometer was removed on a provided log sheet. MVPA levels were assessed subjectively method through an item from the CAPL-2U questionnaire (22), which asked participants to report the frequency of engaging in MVPA for at least 60 min per week. In this study, the average weekly step count will be referred to as the physical activity level.

#### 2.4.4 Physical literacy level

The physical literacy levels of children are determined through composite scores derived from four domains: Physical Competence, Daily Behavior, Knowledge and Understanding, and Motivation and Confidence. Physical literacy is essential for a child's overall development and well-being. Utilizing the CAPL-2 protocol, educators, researchers, and health professionals can comprehensively assess a child's physical literacy, identify strengths and areas for improvement, and develop targeted interventions to enhance physical activity levels. According to the CAPL-2 manual, physical literacy levels are classified into four categories: Beginning, Progressing, Achieving, and Excelling (8). The CAPL-2 scoring system provides a comprehensive evaluation of a child's physical literacy across each domain, with the Achieving level highlighted as the foundational level recommended for optimal health benefits.

In our study, interpretative categories for each domain were adapted to reflect local demographic characteristics. Age- and sex-specific percentiles for each domain and the overall CAPL-2 score were calculated using Generalized Additive Models for Location, Scale, and Shape (GAMLSS), based on data from the Pakistani population. These percentiles determine the child's progression toward achieving the recommended physical literacy levels. The results are interpreted across the four categories as detailed in Table 1. The holistic approach of CAPL-2 ensures that all aspects of physical literacy are measured, providing a detailed understanding of a child's ability to lead an active, healthy lifestyle.

**Table 1:** The CAPL-2 percentile ranges for age and sex specific scores generated for PL and each domain.

CAPL-2 percentile ranges	Categories
Less than 17th percentile	Beginning
Above the 17th–65th percentile	Progressing
Above the 65th–85th percentile	Achieving
Above the 85th percentile	Excelling

#### 2.4.5 Statistical analysis

Data analysis was performed using IBM SPSS Version 22. Descriptive statistics, including means, standard deviations, and percentages, were used to characterize all variables. Outliers were identified through a standardized variable technique, applying *Z*-scores of ±5. Data normality was checked using Q-Q plots and histograms. To examine gender differences in age, height, weight, WC, and CAPL-2 domain scores, we conducted independent samples *t*-tests. Chi-squared tests compared categorical characteristics such as sex, BMI categories etc. Pearson's correlation assessed the relationship among different variables and to further identify variables that will be used in the logistic and regression models. MANOVA was employed to obtained effect sizes and to compare weight statuses across various variables. Binary logistic regression, with the weight statues as the dependent variable was used to determine the odds ratios (with a 95% confidence interval) for being overweight vs. normal weight and the odd ratio was adjusted for other factors Age, Gender, Height, WC, CRF, PA (Number of Steps), MVPA, BD and PL scores.

Structural Equation Modeling (SEM) analyses were conducted using Amos 20.2, with an alpha threshold of 0.05 for statistical significance. The significance of path coefficients was assessed using the critical ratio test, with values over 1.96 indicating significance at the 0.05 level. Statistical significance was established at a p-value of less than 0.05. Effect sizes were classified as small (<0.01), medium (<0.06), or large (>0.14), and Cohen's d calculated the effect size between low and high CRF groups.

**Figure 4:**
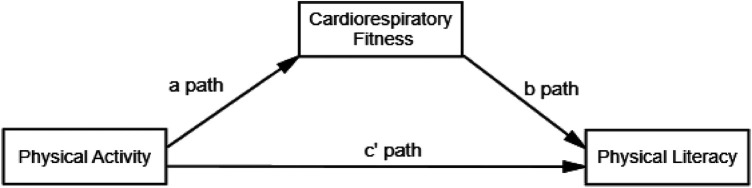
Hypothesized model for the association between the study variables. The figure illustrates the theorized model with physical literacy serving as an outcome.

**H1**: CRF mediates the relationship between PA and Physical literacy level of 8–12-year-old school children. Figure 4 illustrates the relationship between models.

## 3 Results

Table 2 presents descriptive statistics for 1,360 study participants (675 boys, 685 girls) aged 8–12 years. The mean age was 10.00 ± 1.41 years. Physical characteristics: mean height 137.26 cm, weight 30.58 kg, BMI 16.05 kg/m^2^. Weight status by BMI: 5% lean, −79.8% normal weight, 8.7% overweight, 7.1% obese. Notably, more boys had normal weight, while a higher percentage of girls (16.3%) were overweight or obese compared to boys. The mean number of laps completed in the 20 m shuttle test was 19.90 ± 8.04, while the scores achieved in the same test were 3.58 ± 1.63. Boys exhibited higher performance in terms of both laps completed and scores obtained compared to girls in the CRF measure. Boys also took more steps per week (mean: 7,202.28 ± 1,362.02), indicating higher physical activity levels. Boys spent more time in MVPA (mean: 3.89 ± 0.96) and achieved higher scores in daily behavior and Physical Literacy (mean: 10.81 ± 3.04 and 51.15 ± 10.44, respectively) and this gender-based difference was statistically significant (*p* < 0.001). These findings highlight the significant role of gender in physical strength, activity levels, and overall physical literacy.

**Table 2:** Characteristics of the study participants.

Characteristics	Boys (*n *= 675) x̄ ± SD	Girls (*n *= 685) x̄ ± SD	*p*-value
Gender	49.63%	50.36%	–
Age, (years)	10.00 ± 1.42	10.00 ± 1.41	0.908
Height, (m)	137.79 ± 11.42	136.74 ± 11.25	0.088
Body weight, (kg)	30.82 ± 8.82	30.34 ± 8.41	0.308
BMI, (kg/m^2^)	16.04 ± 3.19	16.06 ± 3.14	0.920
BMI classes (CDC)			
Underweight (<5th) %	32 (4.8)	37 (5.5)	–
Normal weight (≥5th–<85th) %	546 (80.9)	539 (78.7)	–
Overweight (≥85th–<95th) %	52 (7.8)	61 (8.9)	–
Obesity (≥95th) %	42 (6.2)	51 (7.4)	–
CRF (laps)	22.61 ± 7.59	17.22 ± 7.57	<0.001
20 m PACER (laps) score	4.14 ± 1.52	3.04 ± 1.55	<0.001
PA (Average weekly steps)	8,136.48 ± 2,486.48	7,589.50 ± 1,789.66	<0.001
MVPA (Self-reported days)	5.17 ± 1.07	4.79 ± 1.09	<0.001
DB scores	12.89 ± 5.22	11.47 ± 3.81	<0.001
PL scores	53.57 ± 9.94	48.58 ± 9.85	<0.001

Data is presented as x¯: mean; SD, standard deviation (or percentages, as indicated); BMI, body mass index; CRF, cardiorespiratory fitness; MVPA, moderate to vigorous physical activity; DB, daily behavior; PL, physical literacy; *p*-value significant at <0.05.

The scatter plot attached as Appendix A presents the relationship between Physical Literacy Scores and cardiovascular fitness, as measured by the Pacer test (expressed as CRF, 20 m PACER Laps scores). The distribution of data points suggests a moderate positive correlation between increased PL and higher CRF level. This is supported by the coefficients of determination for the linear (R^2^ = 0.366) and quadratic (R^2^ = 0.367) models, indicating that approximately 36.6%–36.7% of the variance in PACER scores can be explained by the Physical Literacy Scores.

The results presented in Table 3 demonstrate the relationship between the CRF and various variables, including BMI, Physical Activity, MVPA, Daily Behavior, and Physical Literacy scores. The CRF scores exhibited significant negative associations with BMI and positive associations with PA, MVPA, DB, and PL, as indicated by r values ranging from 0.241 to 0.624. Similarly, BMI displayed significant negative associations with PA, MVPA, DB, and PL scores, with r values ranging from −0.139 to −0.209. In terms of the correlation between subjectively and objectively measured parameters of PA and MVPA, a low to moderate significant positive correlation was observed, with r values ranging from 0.210 to 0.503. Furthermore, objectively measured PA scores demonstrated a strong correlation with the composite scores of DB and PL, characterized by r values between 0.951 and 0.734. Lastly, a noteworthy and strong positive correlation (*r* = 0.775) was identified between composite DB and PL scores.

**Table 3:** Correlation of the CRF with BMI, PA, MVPA, DB, and PL scores.

	CRF scores	BMI (kg/m^2^)	MVPA (Self reported days)	PA (Average weekly steps)	DB scores
BMI (kg/m^2^)	−0.143^a^	–			
MVPA (Self-reported days)	0.241^a^	−0.139^a^	–		
PA (Average weekly steps)	0.486^a^	−0.167^a^	0.210^a^	–	
DB scores	0.506^a^	−0.192^a^	0.503^a^	0.951^a^	–
PL scores	0.624^a^	−0.209^a^	0.399^a^	0.734^a^	0.775^a^

CRF, cardiorespiratory fitness; DB, daily behavior; PL, physical literacy; BMI, body mass index (kg/m^2^); PA, physical activity.

^a^ Correlation is significant at the 0.05 level (2-tailed).

Table 4 shows the binary logistic regression among the normal weight and overweight/obese children. The majority of the children were of normal weight (*n* = 1,085), with a similar gender distribution. Overweight/obese children had significantly higher measurements in height, weight, BMI, and WC (*p* < 0.001). Children of normal weight also had significantly higher levels of physical activity, taking an average of 7,302.52 steps per week and engaging in 5.04 days of MVPA per week, both with p-values less than 0.001. This suggests a strong correlation between higher physical activity levels and normal weight status in children.

**Table 4:** Binary logistic regression among the normal weight and overweight children.

Characteristics		Weight status categories		* *	B	
		NW (1,085)	OW (206)	*η*_p_^2^	OR+ [95% CI]	*P*-value
Age, (years)		9.87 ± 1.41	10.66 ± 1.31*	0.042	0.317 1.373 (1.159–1.627)	<0.001
Gender	Boys	546 (50.3%)	102 (49.5%)		0.115 1.122 (0.726–1.733)	0.604
	Girls	539 (49.7%)	104 (50.5%)			
Height (m)		136.28 ± 11.16	141.09 ± 11.22*	–		
Body weight, (kg)		28.86 ± 6.73	42.72 ± 7.50*	–		
BMI (kg/m^2^)		15.39 ± 2.06	21.34 ± 1.74*	–		
WC (cm)		58.33 ± 8.19	65.41 ± 9.10*	0.088	0.064 1.066 (1.040–1.092)	<0.001
CRF (20 m PACER laps)		20.47 ± 8.19	17.13 ± 6.68*	0.023	−0.051 0.985 (0.951–1.020)	<0.05
PA (Average weekly steps)		7,302.52 ± 1,403.63	6,688.38 ± 926.44*	0.027	−0.004 0.996 (0.995–0.997)	<0.001
MVPA (Self-reported days)		5.04 ± 1.09	4.66 ± 1.14*	0.016	−1.114 0.328 (0.182, 0.593)	<0.001
DB scores		11.06 ± 3.09	9.48 ± 2.28*	0.037	−1.240 3.456 (1.881, 6.351)	<0.001
PL scores		52.08 ± 10.36	46.25 ± 9.52*	0.042	−0.009 0.992 (0.963, 1.021)	0.570
Nagelkerke R square						0.492

Data is presented as x¯ mean, SD, standard deviation; NW, normal weight; OW, overweight/obese; BMI, body mass index; WC, waist circumference; CRF, cardiorespiratory fitness; MVPA, moderate to vigorous physical activity; DB, daily behavior; PL, physical literacy. Dependent variable, weight status; OR, the odd ratio was adjusted for other factors with normal weight as the reference category; *p*-value significant at <0.05; η_p_^2^, partial eta squared; Effect sizes are considered small if <0.01, a medium effect <0.06, and a large effect >0.14.

* Shows significant difference among weight across different variables at *p*-value <0.001.

Better cardiorespiratory fitness is associated with lower odds of being overweight/obese, although the association is not strongly significant (OR = 0.985, *p* < 0.05). Higher average weekly steps are associated with lower odds of being overweight/obese. This result is statistically significant (OR = 0.996, *p* < 0.001). More self-reported days of MVPA are associated with lower odds of being overweight/obese. This result is statistically significant (OR = 0.328, *p* < 0.001). Higher daily behavior scores are associated with higher odds of being normal weight. This result is statistically significant (OR = 3.456, *p* < 0.001). Physical literacy scores are not significantly associated with weight status. The odds of being overweight/obese do not significantly differ based on PL scores (OR = 0.992, *p* = 0.570). This finding highlights the crucial role of physical literacy in promoting a healthy weight among children.

**Figure 5:**
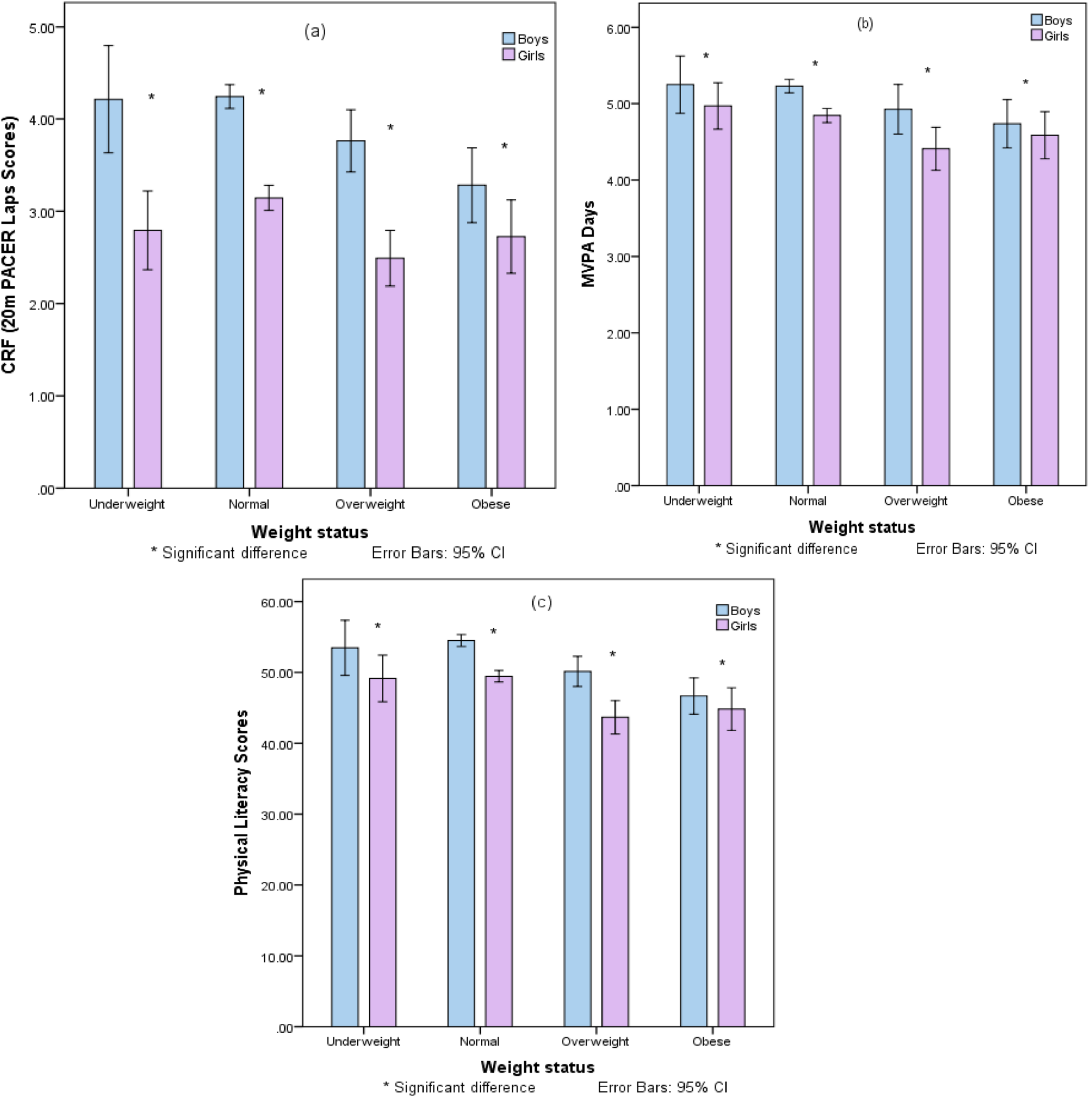
Gender disparities in physical fitness indicators across BMI categories: A comparative analysis of PACER scores, physical literacy, handgrip strength, and MVPA days.

Figure 5 presents bar graphs that compare gender differences in PACER test scores, Physical Literacy Scores, and MVPA days, stratified by BMI categories (Underweight, Normal weight, Overweight, Obese). Boys consistently outperform girls in CRF (20 m PACER laps scores) across all BMI categories, with significantly higher scores observed for boys in each category. Boys also report significantly more MVPA days than girls in every BMI category, indicating higher levels of physical activity. Similarly, boys show significantly higher Physical Literacy Scores than girls across all weight statuses. These findings highlight notable gender disparities in physical fitness indicators among school-aged children in Pakistan, with boys demonstrating superior performance in CRF, MVPA days, and physical literacy. The data, presented with 95% confidence intervals, underscores the need for interventions to promote physical fitness equally among both genders.

### 3.1 Gender-specific associations among cardiorespiratory fitness and physical literacy in children

Tables 5–9 present the associations between the cardiovascular fitness levels and various components of physical literacy across different age groups (8–12 years old) and genders in school children. There is a significant positive association between CRF and PL composite scores, with effect sizes increasing with age, suggesting that the relationship between CRF and PL strengthens as children grow. Consistently, the Physical Competence domain composite score displayed one of the largest effect sizes across age and gender groups, indicating a strong association between CRF and physical skills and abilities. In 8-year-olds (Table 5), boys and girls both showed significant relationships between CRF and PL, with effect sizes ranging from n_p_^2^ = .022 to .490 for boys and n_p_^2^ = .071 to .785 for girls. Notably, the PC domain and the PACER scores for boys (n_p_^2^ = .614 and .628) and girls (n_p_^2^ = .780 and .785) had the largest effect sizes, indicating strong associations with CRF. By age 9 (Table 6), the PC domain composite scores and PACER tests continued to demonstrate strong associations with CRF in boys (n_p_^2^ = .482 and .790) and girls (n_p_^2^ = .493 and .750). The PL composite scores for both genders also showed considerable effect sizes (n_p_^2^ = .105 for boys and n_p_^2^ = .109 for girls).

**Table 5:** Analysis of variance of CRF and PL components in 8-year-old school children, by gender and CRF level.

Variables	Boys					Girls				
	Beginning CRF (5)	Progressing CRF (98)	Achieving CRF (20)	Excelling CRF (12)	*F* (n_p_^2^)	Beginning CRF (17)	Progressing CRF (79)	Achieving CRF (34)	Excelling CRF (7)	*F* (n_p_^2^)
	Mean ± SD	Mean ± SD	Mean ± SD	Mean ± SD		Mean ± SD	Mean ± SD	Mean ± SD	Mean ± SD	
PC domain composite score	15.37 ± 1.42	15.47 ± 2.97	17.97 ± 2.98**	20.14 ± 1.69***	13.05**** (.230)	8.31 ± 2.14	12.66 ± 3.44*	16.98 ± 2.89**	20.36 ± 2.02***	42.54**** (.490)
CAMSA test score	12.25 ± 4.11	12.59 ± 2.51	12.40 ± 3.45	13.92 ± 2.31	0.99**** (.022)	8.59 ± 1.54	9.93 ± 2.36*	12.12 ± 2.73**	13.00 ± 3.61***	12.06**** (.214)
PACER laps (20 m)	12.60 ± 2.08	17.78 ± 4.81*	27.91 ± 1.63**	33.42 ± 4.29***	69.55**** (.614)	6.83 ± 1.24	13.12 ± 4.21*	24.06 ± 2.92**	33.29 ± 4.79	157.48**** (.780)
PACER laps score	2.00 ± 0.01	3.18 ± 0.95*	4.73 ± 0.94**	6.25 ± 0.75***	73.58**** (.628)	1.00 ± 0.01	2.18 ± 0.89*	4.43 ± 0.56**	6.43 ± 0.79***	161.89**** (.785)
Plank score (s)	9.00 ± 1.41	7.80 ± 2.59	8.82 ± 2.20	8.92 ± 1.93	1.60 (.035)	4.24 ± 2.14	6.94 ± 2.95*	8.23 ± 2.73**	9.29 ± 1.25***	9.46**** (.176)
DB domain composite score	14.75 ± 6.18	11.40 ± 2.10*	11.37 ± 0.90**	13.42 ± 5.28	3.65**** (.077)	11.48 ± 0.51	10.39 ± 1.29*	10.89 ± 0.96	11.57 ± 1.13	6.17**** (.122)
Average daily steps Score (weekly)	10.25 ± 6.50	7.31 ± 1.89*	7.23 ± 0.43**	9.41 ± 4.96	4.08**** (.086)	7.12 ± 0.33	7.00 ± 0.85	7.06 ± 0.24	7.29 ± 0.49	0.48 (.011)
MVPA score (self-reported)	4.50 ± 0.58	4.09 ± 0.94	4.09 ± 0.92	4.08 ± 1.00	0.22 (.005)	4.36 ± 0.49	3.40 ± 0.96*	3.83 ± 0.89**	4.29 ± 0.76	7.12**** (.139)
K&U domain composite score	6.75 ± 1.26	6.15 ± 1.57	6.28 ± 1.32	5.58 ± 1.08	0.73 (.017)	5.24 ± 1.71	5.98 ± 1.74	6.69 ± 1.62**	6.00 ± 1.83	2.83**** (.060)
M&C domain composite score	23.32 ± 6.23	17.87 ± 5.98	16.75 ± 5.32	19.17 ± 5.45	0.91 (.020)	17.93 ± 5.33	17.12 ± 5.39	17.81 ± 5.79	15.64 ± 3.94	0.37 (.008)
Physical literacy scores	58.00 ± 6.32	50.54 ± 7.47	52.00 ± 6.61	57.08 ± 7.80	3.32**** (.071)	42.29 ± 6.35	45.50 ± 4.74	51.26 ± 7.6**	52.72 ± 4.54***	8.00**** (.153)

Data is presented as x¯ mean, SD, standard deviation; PC, physical competence; DB, daily behaviors; PACER, progressive aerobic cardiovascular endurance run; CAMSA, Canadian agility and movement skill assessment; CRF, cardiorespiratory fitness; K&U, knowledge and understanding; M&C, motivation and confidence; n_p_^2^, partial eta squared was used to calculate the effect size between the groups of cardiorespiratory fitness levels. The ANOVA test was used to assess the difference between groups; Pairwise comparison.

* *p* < 0.05 for beginning vs. progressing.

** *p* < 0.05 for beginning vs. achieving CRF.

*** *p* < 0.05 for Beginning CRF vs. excelling CRF.

**** *p* < 0.05 for main effect of tests of between-subjects effects.

**Table 6:** Analysis of variance of CRF and PL components in 9-year-old school children, by gender and CRF level.

Variables	Boys					Girls				
	Beginning (20)	Progressing (86)	Achieving (20)	Excelling (9)	*F* (n_p_^2^)	Beginning (3)	Progressing (92)	Achieving (31)	Excelling (11)	*F* (n_p_^2^)
	Mean ± SD	Mean ± SD	Mean ± SD	Mean ± SD		Mean ± SD	Mean ± SD	Mean ± SD	Mean ± SD	
PC domain composite score	12.66 ± 3.09	17.04 ± 2.64*	20.08 ± 1.84**	22.34 ± 1.00***	40.58**** (.482)	8.80 ± 6.04	11.87 ± 3.75*	17.89 ± 3.11**	20.77 ± 2.34***	43.06**** (.493)
CAMSA test score	11.27 ± 2.75	12.68 ± 2.79*	13.10 ± 2.29**	14.33 ± 1.22***	4.44**** (.092)	9.25 ± 0.96	9.56 ± 2.44	13.41 ± 3.43**	14.23 ± 3.90***	19.20**** (.302)
PACER laps (20 m)	10.09 ± 4.00	21.37 ± 4.50*	31.80 ± 1.67**	38.11 ± 3.89***	137.77**** (.759)	7.25 ± 3.59	11.03 ± 3.41*	23.67 ± 3.66**	33.54 ± 2.22***	123.44**** (.736)
PACER laps score	1.77 ± 0.75	3.94 ± 0.83*	6.00 ± 0.01**	7.33 ± 0.71***	164.72**** (.790)	0.75 ± 0.96	1.81 ± 0.75*	4.32 ± 0.54**	6.38 ± 0.51***	133.12**** (.750)
Plank score (s)	6.86 ± 2.23	8.57 ± 1.90*	9.40 ± 1.35**	9.89 ± 0.33***	7.20**** (.142)	4.75 ± 5.50	6.65 ± 3.13*	8.77 ± 2.54**	9.31 ± 1.49***	10.57**** (.193)
DB domain composite score	12.50 ± 5.01	12.36 ± 3.95	13.15 ± 5.27	11.00 ± 1.32	0.55 (.012)	12.50 ± 7.19	11.06 ± 2.37	11.32 ± 2.84	11.15 ± 4.06	1.67 (.036)
Average daily steps score (weekly)	8.36 ± 4.81	8.15 ± 3.76	8.90 ± 4.85	7.22 ± 0.44	0.40 (.009)	9.25 ± 6.65	7.17 ± 2.14	7.42 ± 2.31	8.23 ± 3.27	0.31 (.007)
MVPA score (self-reported)	4.14 ± 0.89	4.20 ± 0.97	4.25 ± 0.85	3.78 ± 1.09	0.59 (.013)	2.75 ± 0.96	3.88 ± 0.95	3.90 ± 1.08	2.92 ± 1.44	5.92**** (.118)
K&U domain composite score	6.27 ± 1.55	6.50 ± 1.59	6.05 ± 1.47	5.67 ± 1.32	1.01 (.023)	7.00 ± 1.41	6.43 ± 1.62	6.00 ± 1.46	5.77 ± 1.64	1.05 (.023)
M&C domain composite score	17.94 ± 6.31	18.50 ± 5.81	18.32 ± 5.78	15.63 ± 5.68	0.83 (.019)	17.25 ± 6.17	17.46 ± 5.63	16.16 ± 5.32	18.16 ± 5.00	1.15 (.025)
Physical literacy scores	48.27 ± 8.89	52.94 ± 7.43*	56.85 ± 8.3**	55.23 ± 5.85***	5.14**** (.105)	41.50 ± 7.19	45.89 ± 7.25	50.19 ± 7.50	55.08 ± 7.45***	5.41**** (.109)

Data is presented as x¯ mean, SD, standard deviation; PC, Physical Competence; DB, daily behaviors; PACER, progressive aerobic cardiovascular endurance run; CAMSA, Canadian agility and movement skill assessment; CRF, cardiorespiratory fitness; K&U, knowledge and understanding; M&C, motivation and confidence; n_p_^2^, partial eta squared was used to calculate the effect size between the groups of cardiorespiratory fitness levels. The ANOVA test was used to assess the difference between groups; Pairwise comparison.

* *p* < 0.05 for beginning vs. progressing.

** *p* < 0.05 for beginning vs. achieving CRF.

*** *p* < 0.05 for beginning CRF vs. excelling CRF.

**** *p* < 0.05 for main effect of tests of between-subjects effects.

**Table 7:** Analysis of variance of CRF and PL components in 10-year-old school children, by gender and CRF level.

Variables	Boys					Girls				
	Beginning (19)	Progressing (67)	Achieving (43)	Excelling (6)	*F* (n_p_^2^)	Beginning (11)	Progressing (76)	Achieving (40)	Excelling (10)	*F* (n_p_^2^)
	Mean ± SD	Mean ± SD	Mean ± SD	Mean ± SD		Mean ± SD	Mean ± SD	Mean ± SD	Mean ± SD	
PC domain composite score	14.43 ± 2.39	16.50 ± 2.02*	19.20 ± 1.81**	22.96 ± 1.49***	46.81**** (.517)	10.61 ± 3.57	14.04 ± 2.37*	16.26 ± 2.34**	19.61 ± 3.02***	29.70**** (.401)
CAMSA test score	11.95 ± 1.84	12.81 ± 2.43	13.55 ± 2.18**	17.17 ± 3.71***	8.27**** (.159)	10.36 ± 1.75	10.47 ± 2.30	11.49 ± 1.92	11.80 ± 1.55	2.43**** (.052)
PACER laps (20 m)	12.58 ± 2.17	19.62 ± 3.48*	29.05 ± 3.14**	35.50 ± 0.55***	188.31**** (.812)	7.64 ± 1.12	15.19 ± 2.58*	23.54 ± 2.21**	30.90 ± 5.59***	197.94**** (.817)
PACER laps score	1.89 ± 0.32	3.51 ± 0.61*	5.43 ± 0.55**	7.00 ± 0.01***	285.37**** (.867)	1.00 ± 0.00	2.58 ± 2.52*	4.38 ± 0.49**	5.80 ± 1.40***	200.68**** (.819)
Plank score (s)	8.26 ± 2.13	8.41 ± 1.95	8.93 ± 1.64	9.83 ± 0.41	1.92 (.042)	5.91 ± 3.27	7.71 ± 2.25*	7.77 ± 2.23**	9.60 ± 1.26***	4.58**** (.094)
DB domain composite score	9.89 ± 1.52	10.97 ± 2.71	16.90 ± 6.65**	25.50 ± 0.84***	36.15**** (.453)	10.45 ± 0.93	10.31 ± 1.47	11.28 ± 2.46	22.60 ± 5.87***	84.24**** (.655)
Average daily steps score (weekly)	6.79 ± 0.92	7.21 ± 2.34	12.33 ± 6.55**	20.83 ± 0.41***	31.70**** (.421)	7.00 ± 0.00	6.58 ± 1.22	7.18 ± 2.38	18.30 ± 5.38***	93.05**** (.677)
MVPA score (self-reported)	3.11 ± 0.99	3.79 ± 0.92*	4.55 ± 0.71**	4.67 ± 0.52***	14.86**** (.254)	3.45 ± 0.93	3.71 ± 0.92	4.10 ± 0.97**	4.30 ± 0.82***	3.00**** (.063)
K&U domain composite score	6.74 ± 1.24	6.44 ± 1.59	6.74 ± 1.36	6.83 ± 0.41	0.65 (.015)	6.73 ± 1.68	6.18 ± 1.63	6.21 ± 1.58	5.60 ± 1.43	0.87 (.019)
M&C domain composite score	12.65 ± 3.63	16.89 ± 4.70*	21.39 ± 5.01**	28.50 ± 0.01***	27.57**** (.387)	12.16 ± 3.37	16.02 ± 3.69*	18.23 ± 4.47**	25.38 ± 4.43***	22.76**** (.339)
Physical literacy scores	45.42 ± 6.53	50.16 ± 7.87*	58.67 ± 8.79**	74.50 ± 5.32***	31.27**** (.417)	37.64 ± 8.52	45.65 ± 7.46*	51.74 ± 7.65**	65.30 ± 9.97***	28.83**** (.394)

Data is presented as x¯ mean, SD, standard deviation; PC, physical competence; DB, daily behaviors; PACER, progressive aerobic cardiovascular endurance run; CAMSA, Canadian agility and movement skill assessment; CRF, cardiorespiratory fitness; K&U, knowledge and understanding; M&C, motivation and confidence; n_p_^2^, partial eta squared was used to calculate the effect size between the groups of cardiorespiratory fitness levels. The ANOVA test was used to assess the difference between groups; Pairwise comparison.

* *p* < 0.05 for beginning vs. progressing.

** *p* < 0.05 for beginning vs. achieving CRF.

*** *p* < 0.05 for beginning CRF vs. excelling CRF.

**** *p* < 0.05 for main effect of tests of between-subjects effects.

**Table 8:** Analysis of variance of CRF and PL components in 11-year-old school children, by gender and CRF level.

Variables	Boys					Girls				
	Beginning (19)	Progressing (57)	Achieving (49)	Excelling (10)	*F* (n_p_^2^)	Beginning (7)	Progressing (78)	Achieving (42)	Excelling (10)	*F* (n_p_^2^)
	Mean ± SD	Mean ± SD	Mean ± SD	Mean ± SD		Mean ± SD	Mean ± SD	Mean ± SD	Mean ± SD	
PC domain composite score	14.32 ± 2.64	16.29 ± 1.99*	19.14 ± 2.29**	22.06 ± 2.34***	41.45**** (.487)	11.04 ± 4.15	13.67 ± 3.07*	16.96 ± 2.91**	20.04 ± 1.59***	29.53**** (.400)
CAMSA test score	11.95 ± 2.55	13.04 ± 2.73	13.14 ± 2.71	16.40 ± 3.37***	5.91**** (.119)	10.25 ± 1.98	10.95 ± 2.68	12.12 ± 2.58**	12.70 ± 1.77***	3.71**** (.077)
PACER laps (20 m)	12.63 ± 3.30	18.91 ± 3.02*	29.22 ± 3.10**	37.70 ± 2.71***	249.17**** (.851)	10.63 ± 7.15	13.72 ± 2.79*	23.59 ± 3.65**	32.50 ± 1.72***	207.84**** (.824)
PACER laps score	2.00 ± 0.58	3.39 ± 0.49*	5.47 ± 0.50**	7.40 ± 0.52***	397.87**** (.901)	1.50 ± 1.41	2.33 ± 0.50*	4.39 ± 0.63**	6.20 ± 0.42***	291.21**** (.868)
Plank score (s)	8.05 ± 2.15	8.25 ± 1.95	8.98 ± 1.74	8.80 ± 2.04	1.78 (.039)	5.88 ± 2.85	7.42 ± 2.57*	8.24 ± 2.31**	9.30 ± 0.95***	5.00**** (.101)
DB domain composite score	10.16 ± 1.74	10.89 ± 2.43	16.35 ± 6.99**	21.90 ± 5.76***	24.54**** (.360)	9.88 ± 1.73	10.46 ± 2.07	13.73 ± 5.77**	20.30 ± 6.50***	21.96**** (.331)
Average daily steps score (weekly)	6.47 ± 1.43	6.72 ± 2.07	12.04 ± 6.52*	17.50 ± 5.28***	26.30**** (.376)	6.50 ± 1.41	7.01 ± 1.57	9.88 ± 5.46**	16.20 ± 6.12***	22.06**** (.332)
MVPA score (self-reported)	3.68 ± 1.00	4.18 ± 0.76*	4.31 ± 0.74**	4.40 ± 0.70***	3.18**** (.068)	3.38 ± 0.74	3.46 ± 0.94	3.85 ± 0.99	4.10 ± 0.99	2.76**** (.059)
K&U domain composite score	6.84 ± 1.50	6.49 ± 1.72	6.49 ± 1.62	6.50 ± 1.90	0.24 (.005)	7.38 ± 1.60	6.29 ± 1.80	6.12 ± 1.82**	7.00 ± 1.89	1.77 (.038)
M&C domain composite score	12.60 ± 3.69	15.51 ± 4.07*	22.29 ± 5.25**	27.39 ± 2.68***	45.48**** (.510)	12.94 ± 3.19	16.12 ± 3.84*	21.00 ± 4.14**	25.35 ± 2.05***	34.08**** (.435)
Physical literacy scores	43.11 ± 7.79	48.58 ± 7.35*	60.6 ± 9.52**	72.00 ± 8.63***	44.60**** (.505)	39.25 ± 8.60	45.22 ± 8.85*	55.93 ± 9.64**	67.90 ± 5.65***	33.23**** (.428)

Data is presented as x¯ mean, SD, standard deviation; PC, physical competence; DB, daily behaviors; PACER, progressive aerobic cardiovascular endurance run; CAMSA, Canadian agility and movement skill assessment; CRF, cardiorespiratory fitness; K&U, knowledge and understanding; M&C, motivation and confidence; n_p_^2^, partial eta squared was used to calculate the effect size between the groups of cardiorespiratory fitness levels. The ANOVA test was used to assess the difference between groups; Pairwise comparison.

* *p* < 0.05 for beginning vs. progressing.

** *p* < 0.05 for beginning vs. achieving CRF.

*** *p* < 0.05 for beginning CRF vs. excelling CRF.

**** *p* < 0.05 for main effect of tests of between-subjects effects.

**Table 9:** Analysis of variance of CRF and PL components in 12-year-old school children, by gender and CRF level.

Variables	Boys					Girls				
	Beginning (11)	Progressing (52)	Achieving (65)	Excelling (7)	*F* (n_p_^2^)	Beginning (16)	Progressing (71)	Achieving (42)	Excelling (8)	*P*-value
	Mean ± SD	Mean ± SD	Mean ± SD	Mean ± SD		Mean ± SD	Mean ± SD	Mean ± SD	Mean ± SD	
PC domain composite score	12.66 ± 2.30	17.80 ± 1.97*	20.02 ± 2.13**	22.80 ± 0.54***	53.93**** (.553)	11.21 ± 3.51	14.16 ± 2.49*	17.97 ± 2.66**	19.52 ± 3.34***	25.22**** (.363)
CAMSA test score	11.00 ± 2.10	13.39 ± 2.99*	14.58 ± 3.12**	15.43 ± 0.53***	5.993**** (.121)	9.85 ± 1.95	10.92 ± 2.64	12.68 ± 2.58**	12.67 ± 4.50	4.43**** (.091)
PACER laps (20 m)	12.18 ± 1.40	20.69 ± 2.86*	29.26 ± 3.27**	38.14 ± 3.76***	183.36**** (.808)	7.92 ± 1.12	14.21 ± 2.90*	26.24 ± 3.71**	33.33 ± 2.25***	82.95**** (.652)
PACER laps score	2.00 ± 0.02	3.75 ± 0.56*	5.45 ± 0.61**	7.29 ± 0.49***	213.08**** (.830)	1.00 ± 0.01	2.44 ± 0.57*	4.83 ± 0.77**	6.50 ± 0.55***	89.43**** (.669)
Plank score (s)	6.73 ± 2.45	9.27 ± 1.18*	9.36 ± 1.21**	10.00 ± 0.01***	14.18**** (.245)	6.69 ± 3.28	7.82 ± 2.25	8.61 ± 2.29**	8.50 ± 2.35	3.08**** (.065)
DB domain composite score	7.18 ± 1.49	10.33 ± 2.98	15.83 ± 6.64**	24.57 ± 0.53***	28.78**** (.397)	8.54 ± 2.26	10.08 ± 1.76	15.07 ± 6.36**	19.50 ± 7.42***	20.54**** (.317)
Average daily steps score (weekly)	4.55 ± 1.97	6.47 ± 2.48	11.65 ± 6.44**	20.00 ± 0.00***	26.53**** (.378)	5.85 ± 1.82	6.45 ± 1.37	11.07 ± 6.13**	15.67 ± 6.71***	19.10**** (.301)
MVPA score (self-reported)	2.64 ± 0.50	3.84 ± 0.90*	4.15 ± 0.79**	4.57 ± 0.53***	12.72**** (.226)	2.69 ± 0.95	3.64 ± 0.90*	4.07 ± 0.75**	3.83 ± 1.17***	6.43**** (.127)
K&U domain composite score	6.64 ± 1.50	6.63 ± 1.61	6.45 ± 1.60**	7.29 ± 1.80	0.54 (.012)	6.46 ± 1.39	6.74 ± 1.37	6.66 ± 1.36	6.50 ± 1.22	0.80 (.018)
M&C domain composite score	17.59 ± 5.65	17.33 ± 4.21	20.41 ± 5.69	26.14 ± 1.85***	9.30**** (.176)	13.13 ± 3.44	17.05 ± 4.42*	22.13 ± 4.33**	23.45 ± 4.43***	19.92**** (.310)
Physical literacy scores	43.82 ± 7.86	51.49 ± 6.11*	59.91 ± 10.60**	75.43 ± 2.64***	29.27**** (.401)	38.46 ± 8.74	46.95 ± 8.09*	58.98 ± 9.34**	63.50 ± 11.10***	23.52**** (.347)

Data is presented as x¯ mean, SD, standard deviation; PC, physical competence; DB, daily behaviors; PACER, progressive aerobic cardiovascular endurance run; CAMSA, Canadian agility and movement skill assessment; CRF, cardiorespiratory fitness; K&U, knowledge and understanding; M&C, motivation and confidence; n_p_^2^, partial eta squared was used to calculate the effect size between the groups of cardiorespiratory fitness levels. The ANOVA test was used to assess the difference between groups; Pairwise comparison.

* *p* < 0.05 for beginning vs. progressing.

** *p* < 0.05 for beginning vs. achieving CRF.

*** *p* < 0.05 for beginning CRF vs. excelling CRF.

**** *p* < 0.05 for main effect of tests of between-subjects effects.

For 10-year-olds (Table 7), there were significant effects across CRF categories for the PACER laps score (n_p_^2^ = .867 for boys and .819 for girls) and the PC domain composite score (n_p_^2^ = .517 for boys and .401 for girls). The effect sizes increased with age, indicating a growing influence of CRF on PL. At age 11 (Table 8), the largest effect sizes were seen in PACER laps score (n_p_^2^ = .901 for boys and .868 for girls) and PC domain composite scores (n_p_^2^ = .487 for boys and .400 for girls), reflecting a consistent pattern of strong association between CRF and these PL components. Finally, for 12-year-olds (Table 9), the PACER laps score effect size reached n_p_^2^ = .830 for boys and .669 for girls. Boys' PL composite scores and PC domain composite scores also had significant effect sizes (n_p_^2^ = .401 and .553), as did the girls' scores (n_p_^2^ = .347 and .363). Across all tables, the Knowledge and Understanding domain showed the lowest effect sizes, suggesting a lesser impact of CRF on this component. Across all age groups, the K&U domain consistently showed the smallest effect sizes, signaling a weaker connection with CRF. The data collectively underscore the substantial role of CRF in influencing physical competence and endurance, with these relationships being statistically significant, strengthening with age, and generally more pronounced in boys than girls.

### 3.2 Mediation analysis

**Figure 6:**
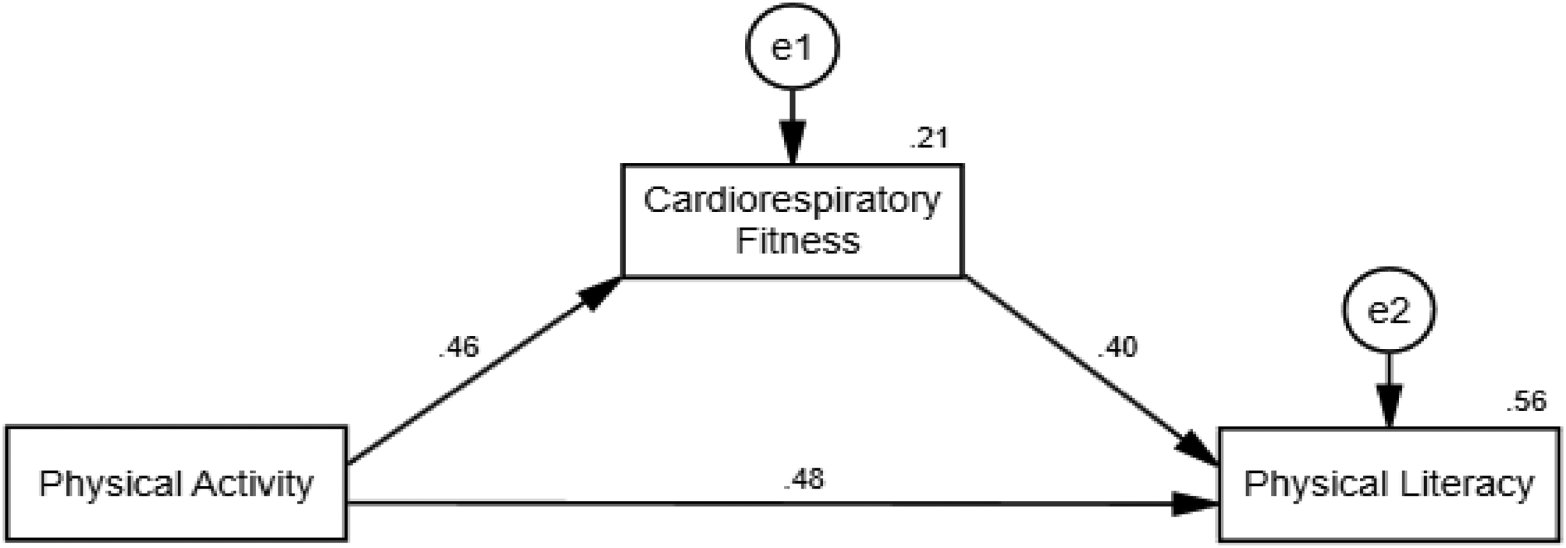
A mediation model of PA and physical literacy relationship medicate by CRF fitness among Pakistani school students.

The mediation role of Cardiorespiratory Fitness on the relationship between Physical Activity and Physical Literacy was evaluated among a cohort of children aged 8–12 years. Structural Equation Modeling was employed, utilizing variance maximum likelihood for parameter estimation. Bootstrap procedures with 2,000 replications were used to provide bias-corrected 95% confidence intervals, thereby enhancing the robustness of the analysis. The multiple mediation model, depicted in Figure 6, was developed to validate the theoretical framework.

The analysis revealed that PA, the independent variable, significantly and positively influenced CRF, the mediator, with a path coefficient of 0.002 (*p* < 0.001). This result indicates that higher levels of physical activity are associated with better cardiorespiratory fitness in children. Subsequently, CRF exhibited a significant positive effect on the dependent variable, Physical Literacy, with a path coefficient of 0.507 (*p* < 0.001), suggesting that improvements in cardiorespiratory fitness correlate with higher levels of physical literacy.

Table 10 shows the results of the modeling of CRF, PA, and PL. Beyond the mediation effects, the direct effect of PA on Physical Literacy was also significant and positive (*β* = 0.002, *p* < 0.001), indicating that PA directly contributes to the improvement of physical literacy levels, independent of the mediating effect of CRF. The indirect effect of PA on Physical Literacy through CRF was significant as well (*β* = 0.001, *p* < 0.001), with bootstrap confidence intervals supporting the mediating effect (LLCI = 0.000, ULCI = 0.002). The absence of zero within the bootstrap confidence intervals confirms the mediating role of CRF. This positive indirect effect highlights the significant role that cardiorespiratory fitness plays as a mediator in the relationship between PA and Physical Literacy.

**Table 10:** Modelling of CRF, MVPA & PL.

Effect type	Relationship	Effect value	Standardized effect	95% confidence interval	*t* statistics
Path a	Physical activity → CRF	0.002*	0.455	[0.001, 0.003]	18.855
Path b	CRF → Physical Literacy	0.507*	0.400	[0.455, 0.559]	19.796
Direct effect c	PA → Physical Literacy	0.002*	0.476	[0.001, 0.003]	23.586
Indirect effect	PA → Physical Literacy	0.001*	0.182	[0.000, 0.002]	–
Total effect	PA → CRF → Physical Literacy	0.003*	0.659	[0.002, 0.004]	15.7021
R^2^ (CRF)			0.207		
R^2^ (PL)			0.560		

Stadarized cofficient reported. Bootstaro Sample: 2,000 with replacement; CRF, cardiorespiratory fitness.

* *p* < value 0.001.

Finally, the total effect of PA on Physical Literacy, which includes both the direct and indirect effects, was substantial (*β* = 0.003, *p* < 0.001). This finding underscores the significant overall impact of PA on physical literacy, demonstrating the combined contribution of PA's direct influence and its indirect influence through improvements in CRF.

## 4 Discussion

This cross-sectional study explores the mediating effect of cardiorespiratory fitness on the relationship between PA and PL in children aged 8–12 from South Punjab, Pakistan. It also examines the correlations among BMI, PA, MVPA, DB, and PL. To our knowledge, this represents the first comprehensive assessment of these variables in the Pakistani and broader South Asian contexts, significantly enhancing our understanding of child health and physical development.

The findings indicate a negative correlation between higher BMI and CRF, highlighting obesity's detrimental effects on physical fitness (3, 30). Previous research by Agostinis-Sobrinho et al. supports this, showing that elevated CRF is associated with lower adiposity and healthier metabolic profiles in school-aged children (31). This is crucial as it points to the challenges faced by children with higher BMI in achieving optimal CRF levels (32). Conversely, the positive correlation between PL and CRF underscores the importance of developing physical literacy as a means to enhance overall fitness and health (3, 33). Notably, CRF correlates significantly with various PL components, particularly physical competence, underscoring its essential role in enhancing children's physical, cognitive, and emotional development (34). Further, a positive association between CRF and both DB and PL scores highlights the broad benefits of CRF, reinforcing its importance in physical health and skill development (3, 6, 35).

### 4.1 Mediation analysis of CRF in the MVPA-Pl relationship

The mediation analysis further explains the role of CRF in the relationship between PA and PL in children. Specifically, higher levels of PA were associated with improved CRF, which in turn was related to enhanced Physical Literacy. These novel findings confirm that engaging in PA enhances PL both directly and indirectly through improvements in CRF, establishing CRF as a pivotal mediator in this relationship. This finding is consistent with previous studies indicating that higher levels of physical activity, leading to improved CRF, can positively influence children's overall physical health (36). Overall, this highlights the importance of PA and CRF for the development of physical literacy and health-related fitness attributes that are crucial during the formative years of children (37). Additionally, monitoring and enhancing CRF are crucial for encouraging physical activity, increasing PL, and detecting cardiovascular diseases and other health abnormalities (38, 39).

Furthermore, the direct impact of PA on PL was both strong and positive, suggesting that physical activity contributes to PL development beyond its influence on fitness. This underscores the continuous role of PA in development of PL in children (40). Considering both direct and mediated paths, the total effect of PA on PL was substantial, emphasizing the essential role of physical activity in children formative years. Evidence indicates that CRF not only facilitates engagement in PA but also enhances PL, advocating for the enhancement of CRF to promote sustained physical activity and advance PL.

Although the relevant research in this domain remains limited, existing studies suggest that the PA-PL relationship during childhood is mediated by health-related fitness factors, particularly CRF (41, 42). Longitudinal studies further support this mediation, reinforcing the significance of CRF in this PA-PL relationship (43, 44). Enhancing CRF and maintaining vigorous physical activity levels can address various health challenges, including issues related to antioxidant capacity and muscle oxygenation (45). Therefore, strategies aimed at boosting CRF may be effective in enhancing overall health and PL and in mitigating obesity's adverse effects in children (30, 46). Importantly, this research highlights the value of incorporating fitness development into educational programs to foster a holistic enhancement of children's physical competencies and knowledge.

### 4.2 Gender disparities in adiposity measures and components of physical literacy

Our findings showed significant gender differences in body composition and physical performance among children in South Punjab. Notably, of the participants, 80% were classified as normal weight, 8.7% as overweight, and 7.1% as obese. Boys mostly fell into the normal weight category, while 16.3% of girls were either overweight or obese. Additionally, the prevalence of overweight and obesity increased with age, aligning with previous studies that estimated 15%-20% obesity rates among South Punjab children, a figure that exceeds national averages (47). This trend, more pronounced in girls as they age, echoes global concerns about childhood obesity, as highlighted by the World Health Organization and studies like Lobstein et al. (48).

In physical measurements, girls demonstrated higher hip circumference and body fat percentages, whereas boys exhibited greater waist circumference and waist-to-hip ratios. Boys also outperformed girls in hand grip strength and the 20 m shuttle test, reflecting superior cardiovascular fitness (49). They engaged in more physical activity, with higher weekly step counts and more MVPA. Consequently, boys attained higher scores in daily behavior and Composite Physical Literacy, revealing significant gender disparities (*p* < 0.001). This trend is consistent with existing research, which suggests that in Pakistan, boys generally enjoy greater freedom and access to participate in physical activities compared to girls (9, 50). This difference in access and societal attitudes towards physical activities for boys and girls contributes to the observed disparities in physical literacy scores (51).

The study provides the foremost evidence from the Pakistani population that boys are generally more physically active than girls, taking more weekly steps and engaging in higher levels of physical activity. This aligns with research by Pojskic and Eslami, who found similar trends in physical activity levels during childhood and adolescence (52). The benefits of such activity include improved CRF, lower BMI, and better scores in daily behavior and physical literacy (49). Furthermore, children with healthier weight statuses and higher physical activity levels had improved physical literacy scores, supporting the positive link between physical literacy, physical activity, and healthier weights (3, 12, 29). This study findings supports existing literature indicating a strong correlation between cardiorespiratory fitness and physical literacy as children age, particularly in physical competence and aerobic endurance, with more pronounced effects in boys (53). Our findings are consistent with prior research demonstrating CRF as a crucial factor in all CAPL-2 domains, correlating with all PL components (12).

The gender-specific analysis revealed a consistent positive association between CRF and PL across age groups, strengthening with age, especially in the physical competence domain (54). This highlights the growing influence of CRF on physical skills as children mature, resonating with findings by Lang et al. regarding CRF's role in developing physical competence (55). Across genders and ages, significant associations were observed in physical competence subdomains, notably in gross motor skills and muscular endurance. This study is pioneering in using CAPL-2 to examine CRF's relationship with motor skills and muscular endurance (56). Other studies corroborate this strong association, employing varied test batteries. Another research also shows a moderate yet significant positive relationship between CRF, the plank test, and muscular endurance, advocating for interventions focused on these areas to enhance a range of physical activities and improve CRF (3).

Our findings reveal a consistent, age-progressive positive association between cardiorespiratory fitness and physical literacy across genders. In line with previous research, the SRT (20 m) is validated as an effective tool for detecting low PL levels, supporting the use of comprehensive CAPL assessments (29). The 20mSRT serves as a practical screening tool in school settings, optimizing resource utilization by efficiently identifying children with low PL. Its application extends to public health surveillance, pinpointing at-risk children (29). This research suggests the development of new criterion-referenced standards for evaluating children's physical literacy across cognitive, mental, and physical health indicators. This could aid in early detection of lifestyle-related disease risks. The study highlights the critical role of CRF in child development and the need for targeted interventions to improve CRF, enhance PL, and maintain overall health. It also emphasizes the need to consider gender differences in intervention design and implementation. Future research should further investigate these relationships to inform policies and practices promoting healthy lifestyles among children.

### 4.3 Study strengths

Methodologically, the study's strength lies in its comprehensive approach, employing a large, diverse sample from South Punjab, Pakistan. Data collection was standardized using the validated CAPL-2 instruments by trained professionals, ensuring reliability across the province (21). Another key strength lies in the use of standardized and robust selection process, ensured a representative sample across various socioeconomic backgrounds, minimizing result disparities (9, 21). This research is pioneering in examining the mediating role of CRF in the relationship between MVPA and PL in a substantial participant group, utilizing CAPL-2, involving a substantial number of participants. This study is pioneering in its examination of the mediating role of CRF between MVPA and PL, supported by both qualitative and quantitative evaluation criteria, which enrich our understanding of the interplay between children's activity levels, fitness, and literacy.

While the use of pedometers to measure physical activity strengthens the study by providing objective data, the discrepancy with accelerometers used in comparative studies should be noted. Future research should explore the longitudinal effects of MVPA on PL and CRF to establish causality and assess the longevity of these impacts. Additionally, identifying which types of MVPA are most effective for enhancing CRF and PL could refine intervention strategies. This study lays the foundation for understanding the interaction between activity, fitness, and literacy, which is crucial for holistic physical development in children. Further investigation into the precision of CRF test thresholds for identifying low physical literacy will advance our understanding in this vital area.

### 4.4 Limitations and implications

This study offers valuable insights, yet its results interpretations must consider certain limitations. The cross-sectional design hinders determining cause-and-effect relationships between CRF, PA, PL, and weight status. Consequently, longitudinal research is required to explain these relationships over time. Further research should expand the structural equation modeling (SEM) to include all four domains of PL, enabling a comprehensive analysis of the factors influencing PL. Such an approach would provide deeper insights, particularly by exploring the interactions between PA, CRF, and the multifaceted aspects of PL labeled as PL1, PL2, PL3, and PL4. Moreover, while the sample size is substantial, its confinement to South Punjab, Pakistan, constrains its generalizability. Future research should encompass a broader demographic range.

The implications of this research are significant. The identified associations among CRF, PA, PL, and weight status highlight the necessity for targeted interventions to combat obesity and promote physical health from an early age. Given the varying impacts across genders, gender-specific strategies may be beneficial. The escalating rates of overweight and obesity with advancing age accentuate the need for early, effective public health initiatives. These should prioritize encouraging physical activity, enhancing CRF, and improving PL among youth to foster better health outcomes.

## 5 Conclusion

This study demonstrated strong associations between cardiorespiratory fitness levels and healthier weight statuses, increased physical activity, and enhanced physical literacy among 8–12-year-old Pakistani children. Our results identify CRF as a crucial determinant of PA and physical literacy. Thus, interventions designed to improve CRF are essential for promoting physical literacy. Additionally, the gender differences observed in CRF highlight the need for tailored strategies in future research. This research highlights the critical role of fostering CRF from a young age, advocating for its integration into school curricula and public health initiatives. Ultimately, this study contributes significantly to the field of children's health by advocating for the promotion of CRF to sustain healthy weight and improve PA and PL levels.

## Data Availability

The raw data supporting the conclusions of this article will be made available by the authors, without undue reservation.
